# Heavy metals in spices from Lancaster, PA: arsenic, cadmium, and lead exposure risks and the need for regulation

**DOI:** 10.1007/s10661-025-14064-9

**Published:** 2025-04-28

**Authors:** Andrew Huff, Hoi Lam, Anuj Ghimirey, Jay Goldring, Michelle Pimentel, Harriet Okatch

**Affiliations:** 1 College of Population Health, Thomas Jefferson University, Philadelphia, PA USA; 2 Touro College of Osteopathic Medicine, New York, NY USA; 3 Kornberg School of Dentistry, Temple University, Philadelphia, PA USA; 4 School of Graduate Studies, Rutgers University, New Brunswick, NJ USA; 5 Public Health Program, Franklin and Marshall College, Lancaster, PA USA

**Keywords:** Heavy metals, Spices, Lead, Arsenic, Cadmium, Regulation

## Abstract

Heavy metal exposure is associated with adverse health effects in children including impaired cognitive functioning. Studies have identified spices as a potential source of heavy metals. However, in Lancaster, PA no such studies have been conducted. Hence the aim of this study was to quantify arsenic (As), cadmium (Cd) and lead (Pb) in spices from Lancaster, PA. Spices were either purchased from stores in Lancaster or obtained as donations from families in Lancaster. As, Cd, and Pb were quantified using inductively coupled plasma-mass spectrometry. The Wilcoxon rank sum test was used to compare the median heavy metal concentrations in store-purchased and home-donated spices. An exposure assessment was conducted to determine the estimated daily intake (EDI) for the metals. As, Cd, and Pb were detected in over 90% of the samples. The As, Cd and Pb median concentrations were 0.048, 0.056 and 0.177 ppm, respectively. 40.5% of the samples exceeded the Pb limit set by the New York State Department of Health; 6.03% and 3.45% of the samples exceeded the As and Cd limits, respectively. Only the As concentration differed significantly (*p* = 0.001) between the store-purchased (0.055 ppm) and home-donated spices (0.034 ppm). The EDI for As, Cd, and Pb based on the median concentrations was 0.016 ug/day, 0.020 ug/day, and 0.062 ug/day, respectively. The elevated lead content in the spices suggests continuous monitoring of heavy metals in spice samples. Standardized guidelines for maximum permissible limits of heavy metals could improve food safety.

**Supplementary Information:**

The online version contains supplementary material available at 10.1007/s10661-025-14064-9.

## Introduction

The World Health Organization (WHO) lists the metalloid Arsenic (As) and the metals Cadmium (Cd) and lead (Pb) among the top ten chemicals of public health concern (World Health Organization, 2020). Even at low concentrations, As, Cd, and Pb (commonly referred to as “heavy metals”) are associated with negative health impacts. For example, As is classified as a carcinogen and higher As concentrations in biological samples are associated with lower cognitive functioning in children (Kaur et al., 2024; McClintock et al., 2012; Vahter et al., 2020). Associations between cognitive impairment and exposure to Cd and Pb have also been reported (Canfield et al., 2003; Kampouri et al., 2024; Lanphear et al., 2005; Stein et al., 2022).

Humans may be exposed to heavy metals through consumption of food (Lasky, 2002). Heavy metal contamination of culinary spices in particular is a global concern, and, in recent years, scholars have approached this topic from multiple angles. Some have focused on the variety of metals found in spices in specific countries, such as Poland (Kowalska, 2021), Bangladesh (Islam et al., 2023), or Ethiopia (Adugna et al., 2024). Others have focused on the variety of metals found in specific spice products, such as powdered ginger (Amponsah et al., 2022), black cumin and fenugreek (Tura et al., 2023), or thyme (Akoury et al., 2022). Meanwhile, in their global systematic review of the levels of heavy metals found in culinary spices, Alawadhi et al. (2024) found variation by both country and spice, underscoring the need for further research grounded in specific local contexts.

In the United States (USA), there are no federal guidelines for acceptable levels of heavy metals in spices, although New York became the first to adopt state-level guidance in 2018 (Ishida et al., 2022). Internationally, the WHO, and countries including China, Malaysia, The Republic of Korea, Singapore and Thailand have set limits for Cd and Pb in herbal remedies (World Health Organization, 2007). While spices are not the only pathway by which heavy metal exposure may occur, it is important to clarify their relative contribution, as it may be large in some cases. For example, in 2021, the Douglas County Health Department in Nebraska reported that 19% of lead exposures were due to contaminated spices, making them the second most common source of lead poisoning (McCracken, 2023).

Previous research in the USA has investigated the levels of Pb in spices (Angelon-Gaetz et al., 2023; Kappel et al., 2021; Napier et al., 2024; Tan et al., 2023). However, research into the levels of other heavy metals in spices is still emerging (Ishida et al., 2022). In Pennsylvania (PA), heavy metal contamination has previously been studied in White-tailed deer (Sileo & Beyer, 1985), plants (Dietterich et al., 2017), water (Dumancas et al., 2022), ambient air (McDonald et al., 2022), and soil (Bassetti et al., 2023), but not spices. Lancaster, PA, is an ideal setting for such a study for two reasons. First, compared to other cities, Lancaster has resettled a higher rate of refugees, who are also known to experience greater risk of lead poisoning compared to the general population (Balza et al., 2023; Seifu et al., 2020; Strasser, 2017). Second, the percentage of children under age six with elevated blood lead levels in Lancaster is nearly twice the overall state percentage (Pennsylvania Department of Health, 2018). As a result, this study aimed to measure the quantities of As, Cd, and Pb in spices obtained from residential and commercial settings in Lancaster, PA.

## Materials and methods

### Study setting and spice samples

The spice samples were either purchased from stores in Lancaster and surrounding areas, or were obtained from homes of multicultural families residing in Lancaster. A total of 116 samples were analyzed; 82 purchased from stores and 34 donated by families. The following information was recorded for the store-purchased spices: spice type, brand name, store name, origin, type of packaging and batch number. This information was not always available for the spices obtained from households. Usually, families donated spices in the original packaging, otherwise the samples were collected in polyethylene bottles (Sigma-Aldrich) certified to have no heavy metal contaminants.

### Heavy metal analysis

Spice samples were shipped to AGQ Labs with chain of custody observed. AGQ Labs is ISO- 17025:2017 accredited by the International Accreditation Service with a scope of accreditation that includes the analysis of heavy metals in food. The samples were analyzed using the Food and Drug Administration Elemental Analysis Manual (FDA EAM) 4.7 method (FDA, US Food and Drug Administration, 2015) for heavy metals in foods using an inductively coupled plasma-mass spectrometry (ICP-MS, Agilent 7900). Briefly, 2 g of the spice samples were homogenized and digested on a hot block with 4 mL nitric acid (Fischer Chemical), 1 mL H_2_O_2_ (Sigma-Aldrich) and 10 mL of water. After digestion, 0.25 mL HCl (Fischer Chemical) was added and made to the 50 mL mark with water.

For quality control, a custom-made certified reference material (CRM) for heavy metals from Inorganic Ventures was used. The CRM was a composite of the following reference materials AGQ 22, AGQ 23, AGQ 29, ACG 23Q, MSHG- 10PPM, and ICP STD Hg 1000 and was used both for initial calibration verification and continuing calibration verification. All the reagents utilized in the analyses were assayed by either ICP-MS or inductively coupled optical emission spectrometry and the trace metallic impurity reported as NA. The multi-elemental standard by Inorganic Ventures was used for the calibration. Linearity, repeatability, spike recovery, limit of detection and limit of quantification calculations, and relative/combined/expanded uncertainty, were conducted according to the internal standard operating procedures (PE- 2118). All water used in these analyses was obtained from the Milli-Q filtration system. The limit of detection for each of the metals was 0.01 ppm.

Metal concentrations below the limit of detection were extrapolated to have a value of LOD/✓2 (Croghan & Egeghy, 2003). Average concentrations and measures of dispersion for each of the metals was presented as median and interquartile range (IQR). Because the data were not normally distributed, the Spearman correlation test was used to investigate the relationship between the metal concentrations in the spices. Wilcoxon rank sum tests were used to test differences between store-purchased and home-donated spices. Differences were considered significant if the *p*-value of the tests was < 0.05. The proportion of samples exceeding the reference values was also recorded. For this work, the standards set by the New York State Department of Health (NYSDOH) for inorganic As, Cd and Pb and the World Health Organization for As, Cd and Pb were used as the reference values (Ishida et al., 2022). All data analysis was performed using Stata Version 14 (StataCorp, 2015).

### Exposure assessment

Spice intake values were derived from the USEPA-WWEIA (United States Environmental Protection Agency - What We Eat In America) Food Commodity Intake Database (FCID) 2005–2010 (*Food Commodity Intake Database*, n.d.) which were in turn calculated from the dietary intake interview component of the National Health and Nutrition Examination Survey (NHANES). This database provides estimates of daily intake of over 500 food commodities expressed as amount consumed per day. Spices included in the analysis were cinnamon, ginger (dried), pepper (black and white), turmeric and spices (other).

Children were selected for this analysis due to their sensitivity to the health effects of heavy metals. The age group 1–5 yr was selected for analysis because children start consuming “adult” food at approximately age 1 and childhood lead screening is recommended between ages 1 and 5 (Wengrovitz & Brown, 2009). The estimated intake for this group of spices in this population was 0.35 g/day or 0.023 mg/kg BW/day using the EPA average body weight of approximately 15 kg for children in this age group (EPA, 2011).

The estimated daily intake of each of the spices was estimated using the equation;

$$\text{EDI}=\left(\text{Cmetal}*\text{IR}\right)/\text{BW}$$

where EDI is estimated daily intake, Cmetal is the metal concentration in ppm, IR is the ingestion rate and BW is the body weight in kilograms.

### Ethical considerations

The research protocol for this study was approved by Institutional Review Board at Franklin and Marshall college. Families who participated completed an informed consent process prior to their participation and received a $25 gift card afterwards.

## Results

### Heavy metal in spices

A total of 116 spice samples were analyzed for three heavy metals of public health concern: As, Cd, and Pb. As, Cd and Pb were detected in 90.37%, 93.1% and 98.3% of the spices, respectively. Seventy percent of the spice samples were purchased from commercial stores; three of the stores explicitly identified as international stores in their names and for two of the stores the names implied they were international. The 82 store-purchased spice samples were identified on the label as masala (*N* = 23, 28.05%), curry powder (*N* = 14, 17.07%), paprika (*N* = 6, 7.32%), turmeric (*N* = 5, 6.10%), anatto (*N* = 4, 4.88%) and other spice samples with low frequencies (*N* = 30, 36.59%). Based on the labels, more than half of the store-purchased samples were either manufactured or processed in India (28.05%) or the USA (23.17%). The other spices originated from the following countries: Pakistan, Nepal, Kenya, Vietnam, Taiwan, Bhutan, Guatemala, Australia, Japan, Thailand and Peru. No information about origin was available for 9.76% of the samples. Overall, the samples were predominantly packaged in plastic (86.59%). The remaining samples were packaged in wax paper (8.54%), glass (3.66%) and cardboard (1.22%) (See Supplemental Table 1).

As shown in Table 1, the highest median concentration was obtained for Pb followed by Cd then As. For As and Pb, the observed median concentration was higher for store-purchased samples than samples donated by families, with the difference statistically significant in the case of As (*p* < 0.001). The highest concentration of As (0.497 ppm) in turmeric from Thailand, Cd (0.294 ppm) in paprika from the USA, and Pb (31.9 ppm) in curry powder from the USA were purchased from the same store whose name implied the store was international (Supplemental Table 1). Weak but significant monotonic relationships were observed for As and Cd concentrations (rs = 0.33, *p* = 0.0003) and for Cd and Pb concentrations (rs = 0.34, *p* = 0.002). However, a moderate and significant relationship was observed for As and Pb concentrations (rs = 0.58, *p* < 0.001).

**Table 1:** Average metal concentration in all spices and by spice source

Metal	All (*N* = 116)		Store-purchased (*N* = 82)	Home donation (*N* = 34)	Wilcoxon rank sum test*
	Median ppm (IQR)	Range ppm	Median ppm (IQR)	Median ppm (IQR)	
Arsenic LOD = 0.010	0.048 (0.028,0.075)	0.007–0.497	0.055 (0.035,0.080)	0.034 (0.019,0.049)	0.001
Cadmium LOD = 0.010	0.056 (0.034,0.083)	0.007–0.333	0.055 (0.037,0.081)	0.058 (0.029,0.092)	0.98
Lead LOD = 0.010	0.177 (0.086,0.339)	0.007–31.9	0.182 (0.105,0.352)	0.119 (0.054,0.279)	0.06

^*^Results were considered statistically significant if the *p*-value was <.05

The proportions of samples exceeding the NYDOH reference level by spice source are summarized in Table 2. Overall, 6.03% of spices exceeded the 2023 reference level for As of 0.21 ppm. A total of 8.54% of store-purchased spices and 0.00% of spices donated by families exceeded this level. With regards to Cd, 3.45% of all samples exceeded the 2023 reference level of 0.26 ppm. When stratified by source of spice, 2.44% of store-purchased spices exceeded the Cd level compared to 5.88% of samples donated by families. Lastly, with regards to Pb, 40.5% of samples exceeded the 2023 reference level of 0.21 ppm. When stratified by source of spice, 43.9% of the store-purchased samples and 32.4% of samples donated by families exceeded this level. The proportion of samples exceeding reference levels varied when the WHO limits for As and toxic metals in herbal medicines and products were applied, in which case fewer samples exceeded the limits—specifically, 0.00%, 1.72% and 0.09% of samples exceeded the limits for As, Cd and Pb, respectively (See Table 2).

**Table 2:** Proportion of samples exceeding the reference levels for all spices and by spice source

Metal	2023 NYDOH Standard (Ishida et al., 2022)	Proportion exceeding reference value (%)			2007 WHO Standard (World Health Organization, 2007)	Proportion exceeding reference value (%)		
		all	stores	homes		all	stores	homes
Arsenic	0.21 ppm	6.03	8.54	0.00	5 ppm*	0.00	0.00	0.00
Cadmium	0.26 ppm	3.45	2.44	5.88	0.3 ppm	1.72	0.00	5.88
Lead	0.21 ppm	40.5	43.9	32.4	10 ppm	0.09	1.22	0.00

^#^The reference levels for As refers to inorganic As

^*^Within this WHO document (World Health Organization, 2007), no standard is explicitly stated for As by WHO. However, other standards, stated but not referenced, from Canada, China, Malaysia, Singapore and Thailand have set the national limits for As at 5, 2, 5, 5, 4 ppm, respectively. We selected to use 5 ppm, the mode from the different limits

### Comparison of As, Cd and Pb in spices originating in the USA vs outside the USA

Sub-analysis of the store-purchased samples revealed that among samples for which country of processing or packaging was known (*N* = 74), the samples from the USA had higher As and Cd concentrations compared to the spices that originated from other countries. The median concentrations for As and Cd for the USA samples were 0.060 ppm and 0.058 ppm, respectively (Table 3). The corresponding values for the spices from the other countries were 0.051 ppm and 0.054 ppm. These differences were not statistically significant: *p* = 0.38 and *p* = 0.84 for As and Cd comparisons, respectively. None of the samples from other countries exceeded the 2023 NYSDOH standard for both As and Cd compared to 1.11% of the USA spice samples that exceed the respective limits. Meanwhile, 47.3% of the samples from outside the USA and 42.1% of the samples from the USA exceeded the NYSDOH limit for Pb of 0.21 ppm. For Pb, the median concentrations were 0.187 ppm and 0.182 ppm for the samples from outside the USA and those from the USA respectively; this difference was not statistically significant (*p* = 0.82).

**Table 3:** Comparison of spices manufactured or packaged in the USA vs. other countries on median concentration and proportion exceeding the NYSDOH standards

Heavy metal	US (*N* = 19)	Other countries (*N* = 55)	*p*-value	US (*N* = 19)	Other countries (*N* = 55)
	Median conc (ppm) (IQR)	Median conc (ppm) IQR	Wilcoxon rank sum test	Exceeding NYSDOH limit, %	Exceeding NYSDOH limit, %
As	0.060 (0.035,0.111)	0.051 (0.037,0.077)	0.38	1.11	0.00
Cd	0.058 (0.026,0.099)	0.054 (0.037,0.069)	0.84	1.11	0.00
Pb	0.182 (0.137,0.336)	0.187 (0.109,0.406)	0.82	42.1	47.3

### Exposure assessment

The estimated EDI values of As, Cd and Pb from the species were calculated for both median and highest metal concentrations and the findings are displayed in Table 4. When considering the median heavy metal concentrations, the EDI values for Pb were higher than those of As and Cd but none of them exceeded the EPA reference doses of 0.6 and 0.54 ug/kg/day for As and Cd, respectively, or the FDA lead reference level of 2.2 ug/day. This trend was also observed when considering the highest heavy metal concentration, except the highest Pb concentration in a curry powder sample exceeded the FDA action level. This is the only sample that exceeded the FDA reference level for Pb. Two samples had EDIs greater than 1 μg/day but less than the action level; a store purchased paprika spice and a home donated spice had EDIs of 1.77 μg/day and 1.41 μg/day, respectively.

**Table 4:** The median and highest estimated daily intake of As, Cd and Pb from the spice samples

Metal	Median concentration (ppm)	Median EDI		Highest concentration (ppm)	Highest EDI	
		μg/kg/day	μg/day		μg/kg/day	μg/day
Arsenic	0.048	0.0011	0.016	0.497	0.0114	0.174
Cadmium	0.056	0.0012	0.020	0.333	0.00766	0.117
Lead	0.177	0.0041	0.062	31.9	0.734	11.2

## Discussion

Our study quantified As, Cd, and Pb in over 90% of spice samples purchased from stores or donated from multicultural families in Lancaster, PA. The heavy metal concentrations determined in our study are similar to those conducted in other settings. A systematic review of 50 studies conducted in different regions of the world—Africa, the Americas, Asia, Europe, and Middle East and North Africa—reported concentrations ranging from not detectable to 2.54 ppm for As, not detectable to 8.07 ppm for Cd, and not detectable to 41.2 ppm for Pb (Alawadhi et al., 2024). Despite a study by Sattar et al., in 1989 that reported detectable levels of Cd (0.65–1.34 ppm) and Pb (6.6–9.2 ppm) in spices (Sattar et al., 1989), heavy metals continue to be detected in spices. Possible sources of Pb in spices include contaminated soil, post-harvest handling processes such as grinding and adulteration. Some studies of metals in spices have displayed higher Pb concentrations relative to As and Cd concentrations (Akhtar et al., 2020; Alhusban et al., 2019). Our findings follow the same trend; over two-fifths of the spice samples exceeded the NYDOH 2023 limit for Pb in spices, whereas less than one-tenth of the samples exceeded the limits for As and Cd.

However, this trend may deviate depending on the standard that is used. Variation exists in the standards that researchers use to determine if heavy metal levels exceed regulations. Typically, the standards referenced have been the “WHO guidelines for assessing quality of herbal medicines with reference to contaminants and residues” (World Health Organization, 2007), “Limit test for heavy metals in food additive specifications” (Joint FAO/WHO Expert Committe on Food Additives (JECFA), 2002), or “Commission Regulation (EU) 2023/915 on maximum levels for certain contaminants in food” (European Commission, 2023). In addition to having different limits for metals, it is noteworthy that these standards were not necessarily developed for spices but rather for other matrices such as herbal medicines and related products, medicinal plants, candy, and therefore force researchers to assume validity when applied to spices. Standards are developed considering both the matrix type and the frequency of use, suggesting that application of a standard developed for a different matrix or food additive to spices might erroneously estimate the level of exposure of heavy metals.

Regulating heavy metal concentrations can be challenging for multiple reasons. As noted, the lack of standards for heavy metal regulation in spices which is further compounded by the fact that spices can be obtained from multiple plant parts such as seed, fruits, bark, and roots; spices derived from the different plant parts might require different standards. Furthermore, some spices such as curry powder exist as a mixture of different spices which adds another layer of consideration in regulation. Additionally, because of globalization and trade, regulation of heavy metals in spices may need a concerted effort. Our findings, consistent with at least one other study (Angelon-Gaetz et al., 2023), revealed higher Pb levels in spices manufactured or processed from other countries compared to the US counterparts. Finally, regulations need to be developed and enforced consistently across all actors in the supply chain. Our study showed that the spices that contained the highest concentration for each of the heavy metals were purchased from the same store. This finding suggests that there may be gaps in the knowledge, attitudes, and practices of business owners related to food safety and heavy metal contamination.

Studies of lead in spices have been conducted in New York and North Carolina (Angelon-Gaetz et al., 2018, 2023; Hore et al., 2019), and lead poisoning investigations in Nevada and Washington identified spices as the source of lead (Kappel et al., 2021; Tan et al., 2023). Furthermore, cases of elevated blood lead levels in children in North Carolina and environmental investigations identified applesauce as the source of lead (Napier et al., 2024). A voluntary nationwide product recall was issued; within 9 months of the first identified cases, 519 cases were reported. Laboratory tests identified cinnamon containing lead chromate as the source of lead in the applesauce. In our study, one spice sample has a concentration that would result in an EDI greater than the FDA reference value for lead. Collectively, these studies suggest the importance of implementing a program to monitor and regulate heavy metals, especially lead, in spices. Currently, such a program does not exist. The use of spices in several foods such as applesauce necessitates monitoring and regulation of heavy metals in spices.

To our knowledge this is the first study of heavy metals in spices conducted in Pennsylvania. In 2018, Pennsylvania was one of three states that had the highest prevalence of childhood elevated blood levels of at least 5 µg/dL (this level has since been changed to 3.5 µg/dL (Ruckart et al., 2021)) in the nation and hence identifying potential sources of lead is prudent (USAFacts team, 2024). Additionally, this study adds to the few studies that have assessed heavy metals in spices in the USA and suggests that spices in the USA may need continuous monitoring for heavy metals. Currently environmental risk assessments for children with elevated blood levels focus on legacy sources of lead such as lead based paint and water. However, given the recent recalls of applesauce (resulting from lead chromate in cinnamon) and the findings of this study, including heavy metal analyses in spices might result in a comprehensive risk assessment and may guide more targeted prevention efforts.

Like the elemental concentrations, the Pb EDI values in this study were higher than those of As and Cd. This has also been observed in other studies (Oladeji et al., 2023). Consumption of these spices at the assumed ingestion rates does not pose a health risk. Nonetheless, the use of heavy metal-contaminated waters for irrigating spices, the natural occurrence of the heavy metals in soils, the frequent use of spices, and adulteration practices such as the addition of lead chromate to spices to enhance color vibrancy and weight (Forsyth et al. 2024), warrant the continuous investigation of heavy metals in spices.

### Limitations

A few limitations need to be considered in the interpretation of the findings of this study. We were unable to obtain complete data (spice type, country of origin and packaging at time of sale) for the spices donated by families and therefore suggest caution in the interpretation of these findings. Additionally, the As standard for NYDOH is specific to inorganic As; our analyses reported total As. Since the total As concentrations of the samples did not exceed the iAs limits, it may suggest that the exposure to iAs from use of these spice samples is minimal. Finally, for the exposure assessment, there were no databases that provided dietary intake values for the exact spices in our study.

## Conclusion

Given the commonplace use of spices in a wide variety of dishes, they represent a viable pathway for chronic, cumulative exposure to heavy metals. In our study we detected As, Cd, and Pb in most of the spice samples. The median concentrations of As, Cd and Pb in the samples were 0.048, 0.056 and 0.177 ppm, respectively, highlighting the need to include spices as part of a wider spectrum of foodborne sources of heavy metal exposure in public health research. The median concentrations were lower than the maximum limits set by the NYSDOH. However, 6.03%, 3.45% and 40.5% of the spices exceeded NYSDOH concentration limits for As, Cd, and Pb, respectively. The corresponding proportion of samples that exceeded the WHO limits were 0.00%, 1.77% and 0.09% for As, Cd, and Pb, respectively. The calculated estimated daily intakes for each of the heavy metals were low and considered to pose minimal risk; however, the lead concentrations of a curry powder sample would result in ingesting lead at levels greater than the FDA reference level.

## Supplementary Information

Below is the link to the electronic supplementary material.

Supplementary file1 (DOCX 32 KB)

## Data Availability

The data utilized in this study are available on request and after consultation with the relevant IRB. De-identified data are included in the Supplemental Table.
