# Supplementary material for: Heavy metals in spices from Lancaster, PA: arsenic, cadmium, and lead exposure risks and the need for regulation
Source: Environ Monit Assess. 2025 Apr 28;197(5):608. doi: 10.1007/s10661-025-14064-9 (PMC12037643; doi:10.1007/s10661-025-14064-9)
Supplement: Supplementary file 1 — Supplementary file1 (DOCX 32 KB) [file 10661_2025_14064_MOESM1_ESM.docx]

Supplemental Table 1: Provides detail for store-purchased spices including the spice name. store ID, type of packaging and heavy metal concentrations.

| **Serial Number** | **Spice Name** | | **Store ID** | **International status** | **Origin** | **Type of packaging** | **AGQ Laboratory Sample Code** | **Total Arsenic (mg/kg)** | **Total Cadmium (mg/kg)** | **Total Lead (mg/kg)** |
| --- | --- | --- | --- | --- | --- | --- | --- | --- | --- | --- |
| 1 | Curry Powder | DV | | No | India | Plastic | AL-20/179912 | 0.093 | 0.052 | 0.258 |
| 2 | Curry Powder | DV | | No | Pakistan | Plastic | AL-20/179913 | 0.097 | 0.037 | 0.155 |
| 3 | Curry Powder | DV | | No | India | Plastic | AL-20/179914 | 0.049 | 0.067 | 0.33 |
| 4 | Pepper | DV | | No | India | Plastic | AL-20/179915 | 0.037 | 0.05 | 0.104 |
| 5 | Turmeric Powder | DV | | No | India | Plastic | AL-20/179916 | 0.026 | 0.011 | 0.082 |
| 6 | Turmeric Powder | TW | | No | N/A | Plastic | AL-20/179917 | 0.017 | 0.007 | 0.032 |
| 7 | Masala | DV | | No | Nepal | Plastic | AL-20/179918 | 0.051 | 0.093 | 0.32 |
| 8 | Blend of Spices | DV | | No | India | Wax paper | AL-20/179919 | 0.042 | 0.055 | 0.142 |
| 9 | Masala | DV | | No | Nepal | Wax paper | AL-20/179920 | 0.071 | 0.109 | 0.611 |
| 10 | Masala | DV | | No | Pakistan | Plastic | AL-20/179921 | 0.074 | 0.03 | 0.11 |
| 11 | Calendula | TW | | No | N/A | Plastic | AL-20/179922 | 0.179 | 0.05 | 0.865 |
| 12 | Annatto | TW | | No | N/A | Plastic | AL-20/179923 | 0.023 | 0.02 | 0.063 |
| 13 | Masala | DV | | No | Pakistan | Plastic | AL-20/179924 | 0.079 | 0.095 | 0.384 |
| 14 | Masala | DV | | No | India | Wax paper | AL-20/179925 | 0.036 | 0.054 | 0.145 |
| 15 | Mustard | HS | | No | N/A | Plastic | AL-20/179926 | 0.019 | 0.234 | 0.007 |
| 16 | Mustard | DV | | No | India | Plastic | AL-20/179927 | 0.013 | 0.08 | 0.013 |
| 17 | Mustard | TW | | No | N/A | Plastic | AL-20/179928 | 0.016 | 0.223 | 0.012 |
| 18 | Guajillg | TW | | No | N/A | Plastic | AL-20/179929 | 0.149 | 0.218 | 0.169 |
| 19 | Biryani spice | DV | | No | Pakistan | Plastic | AL-20/179930 | 0.082 | 0.039 | 0.148 |
| 20 | Paprika | TW | | No | N/A | Plastic | AL-20/179931 | 0.089 | 0.045 | 0.201 |
| 21 | Paprika | DV | | No | Spain or Peru | Plastic | AL-20/179932 | 0.048 | 0.02 | 5.06 |
| 22 | Ancho | TW | | No | N/A | Plastic | AL-20/179933 | 0.223 | 0.224 | 0.284 |
| 23 | Pepper | JJ | | Self-identified | USA | Plastic | AL-20/179934 | 0.057 | 0.075 | 0.286 |
| 24 | Annatto | JJ | | Self-identified | USA | Plastic | AL-20/179935 | 0.015 | 0.013 | 0.137 |
| 25 | Annatto | JJ | | Self-identified | USA | Plastic | AL-20/179936 | 0.035 | 0.03 | 0.251 |
| 26 | Sin achiote | JJ | | Self-identified | USA | Plastic | AL-20/179937 | 0.01 | 0.011 | 0.059 |
| 27 | Salad & vegetable seasoning | JJ | | Self-identified | Puerto Rico | Plastic | AL-20/179938 | 0.007 | 0.007 | 0.174 |
| 28 | Anise seed | JJ | | Self-identified | USA | Plastic | AL-20/179939 | 0.058 | 0.026 | 0.086 |
| 29 | Paprika | JJ | | Self-identified | USA | Plastic | AL-20/179940 | 0.06 | 0.023 | 0.15 |
| 30 | Turmeric Powder | JJ | | Self-identified | USA | Plastic | AL-20/179941 | 0.03 | 0.041 | 0.069 |
| 31 | Paprika | JJ | | Self-identified | USA | Plastic | AL-20/179942 | 0.435 | 0.273 | 0.379 |
| 32 | Honey | RS | | No | Bhutan | Plastic | AL-20/179943 | 0.007 | 0.007 | 0.022 |
| 33 | Old bay seasoning | JJ | | Self-identified | USA | Plastic | AL-20/179944 | 0.048 | 0.058 | 0.182 |
| 34 | Masala | DV | | No | India | Wax paper | AL-20/179945 | 0.039 | 0.044 | 0.157 |
| 35 | Masala | DV | | No | India | Wax paper | AL-20/179946 | 0.028 | 0.053 | 0.099 |
| 36 | Paya | DV | | No | Pakistan | Wax paper | AL-20/179948 | 0.101 | 0.071 | 0.236 |
| 37 | Masala | DV | | No | India | Wax paper | AL-20/179949 | 0.054 | 0.058 | 0.345 |
| 38 | Curry Powder | DV | | No | India | Plastic | AL-20/179950 | 0.035 | 0.047 | 0.105 |
| 39 | Masala | DV | | No | India | Plastic | AL-20/179951 | 0.08 | 0.03 | 0.182 |
| 40 | Fried fish spice | DV | | No | Pakistan | Plastic | AL-20/179952 | 0.107 | 0.04 | 0.192 |
| 41 | Masala | DV | | No | India | Plastic | AL-20/179953 | 0.018 | 0.014 | 0.029 |
| 42 | Classic malt | DV | | No | India | Plastic | AL-20/179954 | 0.007 | 0.007 | 0.027 |
| 43 | Masala | DV | | No | Pakistan | Plastic | AL-20/179955 | 0.052 | 0.028 | 0.109 |
| 44 | Karahi | DV | | No | Pakistan | Plastic | AL-20/179956 | 0.126 | 0.062 | 0.187 |
| 45 | Gourmet mughalai | DV | | No | India | Plastic | AL-20/179957 | 0.077 | 0.045 | 0.413 |
| 46 | Chicken tikka | DV | | No | Pakistan | Plastic | AL-20/179958 | 0.065 | 0.038 | 0.123 |
| 47 | Curry Powder | WB | | Implied by name | USA | Plastic | AL-20/179959 | 0.143 | 0.097 | 31.9 |
| 48 | Curry Powder | WB | | Implied by name | Vietnam | Plastic | AL-20/179960 | 0.068 | 0.052 | 0.335 |
| 49 | Cumin powder | WB | | Implied by name | Taiwan | Glass | AL-20/179961 | 0.277 | 0.033 | 0.352 |
| 50 | Annatto | WB | | Implied by name | Vietnam | Plastic | AL-20/179962 | 0.03 | 0.061 | 0.064 |
| 51 | Paprika | WB | | Implied by name | USA | Plastic | AL-20/179963 | 0.269 | 0.294 | 0.361 |
| 52 | Pepper | WB | | Implied by name | Japan | Glass | AL-20/179964 | 0.038 | 0.184 | 0.099 |
| 53 | Turmeric Powder | WB | | Implied by name | Vietnam | Plastic | AL-20/179965 | 0.442 | 0.067 | 0.543 |
| 54 | Turmeric Powder | WB | | Implied by name | Thailand | Plastic | AL-20/179966 | 0.497 | 0.061 | 2.53 |
| 55 | Masala | PJ | | Self-identified | India | Cardboard | AL-20/179967 | 0.076 | 0.081 | 0.805 |
| 56 | Cardamom | PJ | | Self-identified | Guatemala | Plastic | AL-20/179968 | 0.007 | 0.192 | 0.148 |
| 57 | Mchuzi mix beef flavor | BI | | Self-identified | Kenya | Plastic | AL-20/179969 | 0.007 | 0.007 | 0.027 |
| 58 | Curry powder | BI | | Self-identified | Kenya | Plastic | AL-20/179970 | 0.043 | 0.041 | 0.18 |
| 59 | Curry powder | FL | | No | Australia | Plastic | AL-20/179971 | 0.024 | 0.084 | 0.08 |
| 60 | Yay Spicy Thai | FL | | No | Australia | Plastic | AL-20/179972 | 0.068 | 0.127 | 0.086 |
| 61 | Abuela Mexican | FL | | No | Australia | plastic | AL-20/179973 | 0.22 | 0.149 | 0.507 |
| 62 | Curry powder | FL | | No | USA | plastic | AL-20/179974 | 0.112 | 0.113 | 0.173 |
| 63 | Curry powder | FL | | No | USA | glass | AL-20/179975 | 0.064 | 0.075 | 0.207 |
| 64 | Paprika | FL | | No | USA | plastic | AL-20/179976 | 0.111 | 0.033 | 0.235 |
| 65 | Chunky Chat | GH | | Implied by name | India | Plastic | AL-20/179977 | 0.061 | 0.036 | 0.406 |
| 66 | Masala | GH | | Implied by name | Nepal | Plastic | AL-20/179978 | 0.055 | 0.065 | 0.261 |
| 67 | Curry powder | GH | | Implied by name | Nepal | Plastic | AL-20/179979 | 0.052 | 0.061 | 0.49 |
| 68 | Masala | GH | | Implied by name | India | Plastic | AL-20/179980 | 0.048 | 0.037 | 0.174 |
| 69 | Masala | GH | | Implied by name | India | Plastic | AL-20/179981 | 0.048 | 0.049 | 0.142 |
| 70 | Masala | GH | | Implied by name | India | Plastic | AL-20/179982 | 0.046 | 0.086 | 0.415 |
| 71 | Masala | GH | | Implied by name | Nepal | Plastic | AL-20/179983 | 0.064 | 0.069 | 0.342 |
| 72 | Masala | GH | | Implied by name | Nepal | Plastic | AL-20/179984 | 0.041 | 0.065 | 0.486 |
| 73 | Masala | GH | | Implied by name | India | Plastic | AL-20/179985 | 0.048 | 0.087 | 0.439 |
| 74 | Masala | GH | | Implied by name | Nepal | Plastic | AL-20/179986 | 0.06 | 0.068 | 0.426 |
| 75 | Blend of spices | GH | | Implied by name | India | Plastic | AL-20/179987 | 0.04 | 0.055 | 0.138 |
| 76 | Masala | GH | | Implied by name | Nepal | Plastic | AL-20/179988 | 0.1 | 0.057 | 0.694 |
| 77 | Masala | GH | | Implied by name | India | Plastic | AL-20/179989 | 0.038 | 0.05 | 0.305 |
| 78 | Masala | GH | | Implied by name | Nepal | Plastic | AL-20/179990 | 0.028 | 0.037 | 0.28 |
| 79 | Blend of spices | TE | | No | USA | Plastic | AL-20/179991 | 0.057 | 0.047 | 0.1 |
| 80 | Curry powder | TE | | No | USA | plastic | AL-20/179992 | 0.078 | 0.099 | 0.336 |
| 81 | Curry powder | TE | | No | USA | plastic | AL-20/179993 | 0.071 | 0.061 | 0.151 |
| 82 | Curry powder | TE | | No | USA | plastic | AL-20/179994 | 0.066 | 0.105 | 0.454 |
